# Editorial: Advanced nano-bio interfaces for biosensing and diagnostics

**DOI:** 10.3389/fbioe.2023.1296849

**Published:** 2023-09-29

**Authors:** Yuan Jia, Miao Wang, Haogang Cai

**Affiliations:** ^1^ Sino-German College of Intelligent Manufacturing, Shenzhen Technology University, Shenzhen, China; ^2^ College of Materials, Xiamen University, Xiamen, China; ^3^ Department of Radiology, Tech4Health Institute, NYU Langone Health, New York, NY, United States

**Keywords:** nano-bio interfaces, biosensing, bioelectronics, microfluidics, artificial intelligence

Recent advancements in micro and nanotechnologies have catalyzed a transformative era in nano-bio interfaces, bolstering their applicability in diverse biomedical research fields. These applications incorporate point-of-care diagnostics, precision disease diagnostic tools, and automated lab-on-a-chip devices, all tailored to meet the demands of modern healthcare industry. The integration of nanomaterials with state-of-the-art biosensors provide the advantages of miniaturization and cost-effectiveness, high sensitivity and specificity, as well as the potential for compatible fabrication by semiconductor manufacturing processes. Enables the improvement in sensitivity and specificity. The integrated nano-bio sensors have garnered substantial attention for their role in disease detection, particularly in critical scenarios like COVID-19 and cancer, and in the development of ultra-sensitive biosensors. This research endeavor delves into the realm of “Nano-Bio Interfaces,” presenting a multidisciplinary approach aimed at crafting innovative tools to serve diverse facets of biomedical research. In this Research Topic, the scope spans from the conception and realization of nano-bio interface devices to their pragmatic applications in biomedical research, disease diagnosis, and subsequent clinical deployment. The Research Topic collection includes six articles, four Original Research articles, one Review, and one Mini Review. Together, these contributions collectively advance the frontiers of knowledge in the field of nano-bio interfaces, ushering in novel insights and applications for biomedical research and clinical care.

The original research articles in this Research Topic highlight the incorporation of either nanomaterials or nanoscale structures as essential elements in the development of novel biosensors based on various transduction mechanisms, from electrochemistry, bioelectronics to optics. Specifically, Yaiwong et al. presented an *electrochemical* aptasensor for quantifying alpha-fetoprotein, a critical liver cancer biomarker. What sets this sensor apart is its use of polyethyleneimine-coated gold nanoparticles (PEI-AuNPs) as a fundamental component. These nanoparticles serve as a platform for sensor modification, achieving an impressive linear detection range and a low limit of detection, particularly when applied to human serum samples, thus making it a valuable tool for clinical diagnosis of liver cancer. This underscores the crucial role of nanotechnology in advancing biosensing capabilities for critical biomedical applications.

Smith et al. utilized a lab-on-CMOS *capacitive* biosensor platform to quantitatively track the proliferation of murine macrophages. The study establishes a linear correlation between macrophage proliferation and capacitance growth factor. The significance of the article is it monitors cell proliferation over a wider spatial and temporal scale. This innovative perspective opens up possibilities for broader insights and applications in the realm of nano-bio interfaces, particularly for understanding cell behavior and dynamics in various biomedical contexts. Bioelectronic sensors also use field-effect transistors functionalized by biomolecules, which have been enhanced by the adoption of carbon-based nanomaterials, including graphene, carbon nanotubes, and graphene oxide. Hu et al. provided a mini-review explores the burgeoning field of flexible biosensors, with a specific focus on their integration with graphene field-effect transistors (GFETs). GFETs, characterized by their high sensitivity, multifunctionality, rapid response, and cost-efficiency, serve as integral components in constructing these innovative biosensors. The integration of GFETs on flexible substrates provides the advantages including biocompatibility and adaptability to complex environments. The review provides insights into design strategies for flexible GFET biosensors and highlights recent advancements in broad applications for detecting various biomarkers, such as proteins, glucose, and ions.

Lin et al. presented an *optical* biosensor based on cholesteric liquid crystal (CLC). The LC surfaces were functionalized by antibodies to capture the SARS-CoV-2 spike proteins. The antigen-antibody binding formed an immune complex with a molecular weight sufficient to cause disturbance in the LC molecular arrangement, which changed from flat to focal cone state for an optical read-out. The original LC orientation was controlled by N, N-dimethyl-n-octadecyl-3-aminopropyltrimethoxysilyl chloride alignment layers, which contribute to the accuracy and sensitivity. The biosensor is label-free and amplification-free, where the LC color change can be directly seen by naked eyes, thus making the biosensor promising for the rapid point-of-care testing. Meanwhile, loop-mediated isothermal amplification (LAMP) technique also makes nucleic acid amplification compatible with the point-of-care testing. Dong et al. presented the development of a paper-based nucleic acid amplification tests for the detection of SARS-CoV-2 gene fragments. Combining amplification with fluorescence detection, the microfluidic paper-based devices achieved a practically relevant limit of detection of 1,000 copies/mL while maintaining a minimal reagent consumption of only 2.2 μL per readout, at a low power dissipation of 6.4 W. This innovation holds promise for efficient and resource-sparing pathogen detection, particularly in pandemic control efforts.

With recent progresses, microfluidics have not only improved the resource efficiency, but also become more “intelligent.” Sun et al. provided an overview of the fascinating convergence of droplet microfluidics and deep learning, two cutting-edge fields that have garnered significant attention in recent years. The review highlights the interdisciplinary nature of contemporary research and introduces the concept of “intelligent microfluidics,” which combines microfluidic technology with artificial intelligence. It discusses applications of this fusion, particularly in droplet generation, control, and analysis. It also addresses the challenges and emerging opportunities in the field. Ultimately, this evolving synergy between microfluidics and deep learning holds promise for the development of automated, intelligent devices for the smart and high-precision analysis at nano-bio interfaces.

The works presented in the Research Topic underscores the growing importance of nano-bio interfaces in biomedical applications. From advanced biosensors utilizing nanomaterials to the integration of microfluidics and artificial intelligence, these studies highlight the potential of nanotechnology and interdisciplinary approaches in advancing our understanding of biological systems and enhancing healthcare technologies. Based on the collection of articles, it is evident that nano-bio interfaces will continue to play a pivotal role in the development of innovative tools for diagnostics, disease monitoring, and personalized medicine. The combination of nanoscale materials, advanced sensing techniques, and intelligent systems promises to revolutionize the way we interact with and understand the complexities of biological processes. Future research should focus on addressing challenges such as scalability, clinical validation, and regulatory considerations to ensure the seamless translation of these technologies into practical healthcare solutions.

